# Oncolytic Viruses: Promising Future in Cancer Treatment

**DOI:** 10.34172/apb.025.44052

**Published:** 2025-10-11

**Authors:** Ahmed Naueen Nazim, Kannan Subbaram, Razana Faiz, Sheeza Ali

**Affiliations:** School of Medicine, The Maldives National University, Male’, Maldives

## To Editor,

 Oncolytic viruses (OVs) have been extensively investigated over the past two decades for their potential to treat diverse cancer types.^1^ OVs are naturally occurring or engineered viruses that selectively replicate within neoplastic cells, inducing cell lysis while sparing healthy tissue.^2,3^ OVs can be classified as DNA and RNA viruses based on their genomic composition. DNA OVs are ideal due to the high stability of their polymerase enzyme and high replicative ability. The RNA viruses, due to their small size and ability to cross the blood–brain barrier, are suitable for targeting central nervous system tumors.^2^

 PV-701 (a highly purified isolate of the MK107 vaccine strain of Newcastle disease virus), Seneca Valley virus NTX-010 (a picornavirus) and Reolysin are all intravenously administered naturally occurring OVs.^4^ Most other OVs are oncolytic adenoviruses (e.g., H103, Telomelysin, ONYX-015, AdAPT-001), herpes simplex virus -1 (HSV-1) based viral constructs (e.g., HSV1716, T-VEC), and oncolytic vaccinia viruses (e.g., JX-594).^4^

 To date, only four OVs have been approved for the treatment of cancer. They are: a) ECHO-7/Rigvir (a naturally occurring picornavirus) approved in Latvia in 2004, b) H101/Oncorine (a modified adenovirus) approved in China in 2005, c) T-VEC/talimogene laherparepvec (an attenuated HSV-1) approved by the U.S. Food and Drug Administration in 2015, d) and teserpaturev/G47∆/Dalytact (a triple- mutated HSV-1) which was approved in Japan, in 2021.^2^

**Figure 1:**
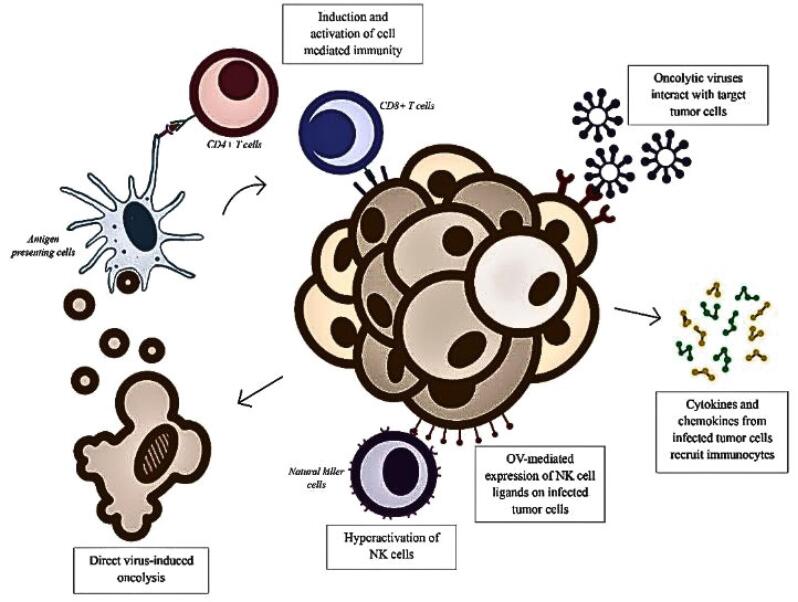
Oncolytic virus-induced cancer cell lysis

 Multiple clinical studies have demonstrated various mechanisms by which oncolytic viruses mediate their antitumor activity, with direct virus-induced oncolysis through invasion and propagation within neoplastic cells being a well-recognized process.^1,3,4^ In addition, studies have shown that induction and facilitation of the innate and adaptive immune responses by OVs play a pivotal role in its antitumor efficacy.^1-3^ A study conducted by Jiang et al in 2024 showed that natural killer cells in combination with oncolytic adenovirus delta24RGD elicited enhanced and sustained long term antitumor effects. These effects were against pancreatic ductal adenocarcinoma (in vitro) and glioblastoma (in vivo). This study also showed that there was increased expression of activating receptors and cytotoxic markers by these cells as well as OV-mediated NK cell ligand expression on infected tumor cells.^5^ Furthermore, infected tumor cells expressed several cytokines including IFN-β, IL-1β, IL-12, IL-4 and TNF-α, and chemokines such as CXCL9, CCL5 and CX3CL1. This has significantly enhanced the chemotaxis and activation of immune cells including CD 8+ T cells^2^ (Figure 1). OVs selectively multiplies in tumor cells resulting in direct cancer cell death and induction of anti-cancer immunity through T-cell and NK cell activity regulating subcellular pathways.^6^ However, studies have indicated that the use OVs have shown limited clinical efficacy than a better preclinical promise. It includes tolerance to immune clearance, in vivo tumor cell entry, and survival under hypoxic conditions.^7,8^

 Numerous studies highlight the therapeutic potential of combining OVs with immunotherapies, including immune checkpoint inhibitors (ICIs) and tumor-infiltrating lymphocytes (TILs).^1^ A study conducted in 2018 by Wing et al using the oncolytic adenovirus Ad-EGFR BiTE showed increased efficacy of CAR-T cells (genetically engineered T cells which express chimeric antigen receptors).^9^ Furthermore, OVs can also activate TILs and enhance their immune response against tumors by facilitating T cell infiltration and activity.^1^ The OV combination therapy has been evaluated to be well tolerated with no exacerbation of expected adverse effects associated with chemoradiotherapy and immunotherapy. Nevertheless, some studies show that children may have higher risk of developing symptoms of pathogenic viral infection, which were generally mild and resolved quickly.^4^ Studies have shown that OVs can exhibit broader safety concerns due to their off-target binding effects, immune escape and may lead to viral shedding.^9,10^ Other studies observed that OVs expressed reduced efficacy on solid tumors, and limited effectiveness in heterogenous tumors. The immune resistance, and tumor micro-environment barriers can be a potential challenge to OV therapy.^7,11^ Studies suggested that OVs in combination with tumor immune therapies like adoptive cell treatments and immunity check-point inhibitors can solve these limitations.^11^ Administering OVs intravenously may reduce overall treatment efficacy, possibly due to active removal of the viruses and binding of viruses to various other host cell types. Therefore, to enhance the antitumor activity in patients with widely disseminated disease, it is crucial to develop oncolytic viruses which can be delivered systemically.^4^ Additionally, further research is warranted to explore proper combination therapies, suitable administration strategies including dosage amounts and timings, and predictive biomarkers corresponding to the tumor response.^1-4^

 The limitations, controversies and unresolved questions on OV application are delivery restrictions, impermeability of DNA OVs passing through blood-brain barrier, and immune escape. Combination of OVs along with chemotherapy, radiotherapy and immune check-point inhibitors can have synergistic effect.

## Conclusion

 The OVs are novel and promising tools for cancer therapy. There are many potential OVs identified for anti-cancer treatment. There are four OVs have been approved that includes, ECHO-7/Rigvir, H101/Oncorine, T-VEC/Talimogene laherparepvec and Teserpaturev/G47∆/Dalytact. OVs acts by selectively targeting cancer cells by direct lysis and inducing anti-cancer immunity. However, many other OVs showed promising testing and limited clinical efficacy. Other limitations of OV usage are delivery challenges, immune clearance, and tumor heterogeneity. Future directions and research are warranted on merging tools like biomarker-guided OV selection or integration with personalized medicine.

## Competing Interests

 Not applicable.

## Consent for Publication

 Not applicable.

## Data Availability Statement

 Not applicable.

## Ethical Approval

 Not applicable.
